# A nomogram predicting 30-day mortality in patients undergoing percutaneous coronary intervention

**DOI:** 10.3389/fcvm.2022.897020

**Published:** 2022-08-17

**Authors:** Jingjing Song, Yupeng Liu, Wenyao Wang, Jing Chen, Jie Yang, Jun Wen, Jun Gao, Chunli Shao, Yi-Da Tang

**Affiliations:** ^1^State Key Laboratory of Cardiovascular Disease, Department of Cardiology, National Center for Cardiovascular Diseases, Fuwai Hospital, Chinese Academy of Medical Sciences and Peking Union Medical College, Beijing, China; ^2^Department of Cardiology, Guangdong Cardiovascular Institute, Guangdong Provincial People’s Hospital, Guangdong Academy of Medical Sciences, Guangzhou, China; ^3^Department of Cardiology, Institute of Vascular Medicine, Peking University Third Hospital, Beijing, China; ^4^Key Laboratory of Molecular Cardiovascular Science, Ministry of Education, Beijing, China

**Keywords:** nomogram, risk prediction, percutaneous coronary intervention, 30-day mortality, model establishment

## Abstract

**Background and aims:**

Early detection of mortality after percutaneous coronary intervention (PCI) is crucial, whereas most risk prediction models are based on outdated cohorts before the year 2000. This study aimed to establish a nomogram predicting 30-day mortality after PCI.

**Materials and methods:**

In total, 10,444 patients undergoing PCI in National Center for Cardiovascular Diseases in China were enrolled to establish a nomogram to predict 30-day mortality after PCI. The nomogram was generated by incorporating parameters selected by logistic regression with the stepwise backward method.

**Results:**

Five features were selected to build the nomogram, including age, male sex, cardiac dysfunction, STEMI, and TIMI 0–2 after PCI. The performance of the nomogram was evaluated, and the area under the curves (AUC) was 0.881 (95% CI: 0.8–0.961). Our nomogram exhibited better performance than a previous risk model (AUC = 0.7, 95% CI: 0.586–0.813) established by Brener et al. The survival curve successfully stratified the patients above and below the median score of 4.

**Conclusion:**

A novel nomogram for predicting 30-day mortality was established in unselected patients undergoing PCI, which may help risk stratification in clinical practice.

## Introduction

Annually, percutaneous coronary intervention (PCI) has been widely used in millions of patients with ischemic heart diseases to save lives and improve the quality of life around the world (1), and the number of patients undergoing PCI is still increasing. However, mortality still exists after PCI, especially in high-risk patients (2). Therefore, it is valuable to develop a risk-prediction model which can help doctors identify patients who are at high-risk and select suitable treatment strategies.

Approximately two-thirds of death after PCI occurs within 30 days after the procedure (3). Therefore, evaluating 30-day mortality after PCI may help identify events related to PCI (4–6). In previous studies, several prediction models have been established to predict the mortality of patients undergoing PCI. However, most of them are based on outdated cohorts in the 1990s (5, 7, 8). In the past two decades, with the progression in intervention strategy, stent technology, and pharmacological therapy, the outcomes of patients undergoing PCI have improved considerably. Therefore, an updated risk prediction model is also needed. Recently, Brener et al. established a novel risk score predicting 30-day mortality after PCI (called Brener’s score hereafter) (9). Brener’s score was derived from 21 randomized PCI trials and showed a good concordance index value of 0.848 for predicting 30-day mortality after PCI. The Brener’s score filled the blank of the risk score predicting 30-day mortality after PCI in the last 15 years. However, Brener’s score lacks data on cardiac function and renal function, which were closely related to mortality after PCI.

Nomogram is a medical tool calculating the risk of a particular clinical outcome for each individual in a graphic manner. It has been applied in risk prediction for cancer, stroke, and surgery (10–12). However, there is no nomogram for predicting the risk of 30-day mortality after PCI. Therefore, this study aimed to establish a novel nomogram for predicting 30-day mortality from a large-scale PCI cohort.

## Materials and methods

### Study population

Our original cohort included 10,724 consecutive patients undergoing PCI at the National Center for Cardiovascular Diseases in China (Fuwai Hospital, Beijing, China) between January 2013 and December 2013. The clinical data were collected prospectively. Patients with age <18 and without left ventricular ejection fraction (LVEF) data were excluded. Finally, we enrolled 10,444 patients. All patients had written informed consent. The study complied with the Declaration of Helsinki and was approved by the Institutional Review Board of the hospital.

### Endpoint and definitions

The primary endpoint was all-cause mortality within 30 days after PCI. Left ventricular function was evaluated by echocardiography before the PCI procedure. Types of stents were recorded in the PCI procedure. Cardiac dysfunction was defined as LVEF <50% (13), and renal insufficiency was defined as the estimated Glomerular filtration rate (eGFR) <30 ml/min/1.73 m^2^ (14). Patients were followed up at a regular interval.

### Statistical analysis

Continuous variables were compared by the Student’s *t*-test, and categorical variables were compared by the chi-square test. Variables in Brener’s model and traditional risk factors were candidates for univariable logistic analysis. Variables with a *p*-value < 0.1 in the univariable logistic analysis were selected for subsequent stepwise backward multivariable logistic analysis, and variables with a *p*-value < 0.05 were retained in the multivariable logistic regression. Then, the nomogram was established based on the results of the multivariable analysis, and the coefficient of each variable was converted into a 0–100-point scale. The area under the curves (AUC) was internally validated by bootstrapping with 1,000 resamples. Calibration plots were generated to evaluate the consistency between nomogram-predicting probability and actual probability. Also, the calibration plots were generated through bootstrapping with 1,000-fold resamples. The AUC was calculated to evaluate the performance of the predictive model. The AUC values between 0.7 and 0.8 are considered moderate, and the AUC values higher than 0.8 are considered good. The survival probability was observed by the Kaplan–Meier analysis. The *p*-value < 0.05 was considered to be statistically significant. The data were analyzed with SPSS version 24.0 (Chicago, IL, United States) and R statistical software version 4.0.3^1^.

## Results

### Baseline characteristics

The baseline characteristics of the patients are shown in Table 1. Among 10,444 patients in our study, the patients who died were significantly older than the living patients and were more likely to be women and smokers (*p* < 0.001). Patients with renal insufficiency, cardiac dysfunction, STEMI, prior MI, TIMI flow 0–1 before PCI, TIMI flow 0–2 after PCI, unstable angina, and second-generation DES implantation had a higher probability of death (all *p* < 0.05). Other characteristics had no significant difference.

**TABLE 1:** Baseline characteristics.

	Alive (*n* = 10419)	Death (*n* = 25)	*P*-value
Age	58.3 (10.3)	69.5 (10.9)	< 0.001
Men	8044 (77.2)	9 (36.0)	< 0.001
Cardiac dysfunction	693 (6.7)	8 (32.0)	< 0.001
Renal insufficiency	284 (2.7)	3 (12.0)	0.026
Diabetes mellitus	3165 (30.4)	4 (16.0)	0.179
Smoking	5950 (57.1)	8 (32.0)	0.02
Hypertension	6701 (64.3)	16 (64.0)	1
Hyperlipidemia	7033 (67.5)	13 (52.0)	0.15
Prior PCI	2568 (24.6)	7 (28.0)	0.876
Prior CABG	1110 (10.7)	5 (20.0)	0.235
Prior Cerebrovascular disease	428 (4.1)	1 (4.0)	1
Prior MI	3765 (36.1)	17 (68.0)	0.002
ACS	6498 (62.4)	17 (68.0)	0.708
Unstable angina	4496 (43.2)	2 (8.0)	0.001
NSTEMI	469 (4.5)	2 (8.0)	0.719
STEMI	1533 (14.7)	13 (52.0)	< 0.001
Stable coronary artery disease	3921 (37.6)	8 (32.0)	0.708
Left main artery disease	650 (6.2)	3 (12.0)	0.438
LM-LAD	7546 (72.4)	16 (64.0)	0.473
Multivessel disease	7840 (75.2)	20 (80.0)	0.75
TIMI flow 0–1 before PCI	2240 (21.5)	10 (40.0)	0.045
TIMI flow 0–2 after PCI	366 (3.5)	6 (24.0)	< 0.001
First-generation DES	995 (9.5)	2 (8.0)	2
Second-generation DES	8945 (85.9)	17 (68.0)	0.023
Lesion length	29.0 (19.3)	31.6 (16.9)	0.497

Variables are shown as mean (standard deviation, SD) or n (%). CABG, coronary artery bypass grafting; ACS, acute coronary syndrome; NSTEMI, non-ST-segment elevation myocardial infarction; STEMI, ST-segment elevation myocardial infarction; LM-LAD, left main coronary artery disease and left anterior descending artery disease; DES, drug-eluting stent; TIMI, thrombolysis in myocardial infarction. Cardiac dysfunction was defined as LVEF <50%. Renal insufficiency was defined as estimated Glomerular filtration rate (eGFR) <30 ml/min/1.73 m^2^.

### Predictors of 30-day mortality after percutaneous coronary intervention

**FIGURE 1:**
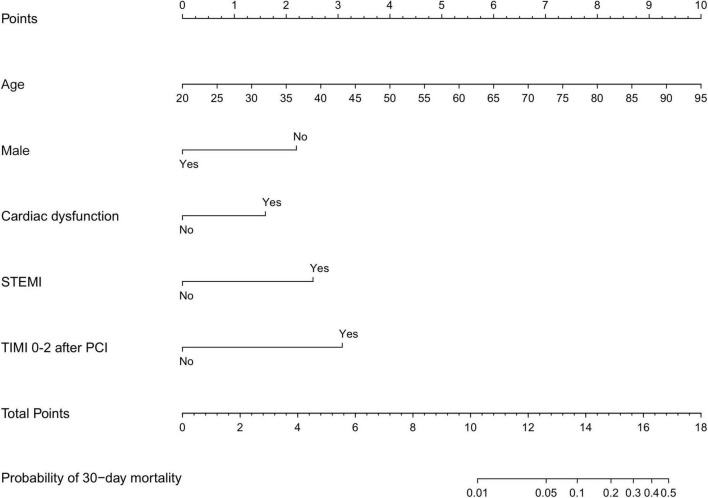
Nomogram scores of 30-day mortality. Nomogram for predicting the probability of 30-day mortality after PCI. Points were assigned for age, male sex, cardiac dysfunction, STEMI, and TIMI 0–2 after PCI by drawing a line upward from the corresponding values to the “points line”. The “total points” are calculated as the sum of the individual score of each of the five variables included in the nomogram. STEMI, ST-segment elevation myocardial infarction; TIMI, thrombolysis in myocardial infarction; PCI, percutaneous coronary intervention. Cardiac dysfunction was defined as LVEF <50%.

**FIGURE 2:**
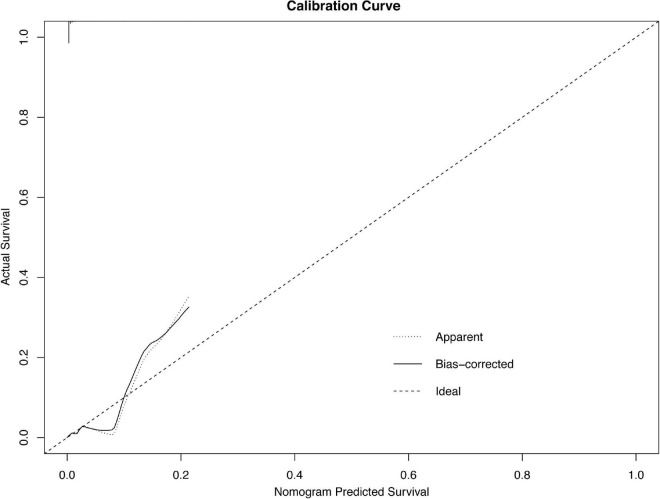
Calibration plots of the nomogram.

**FIGURE 3:**
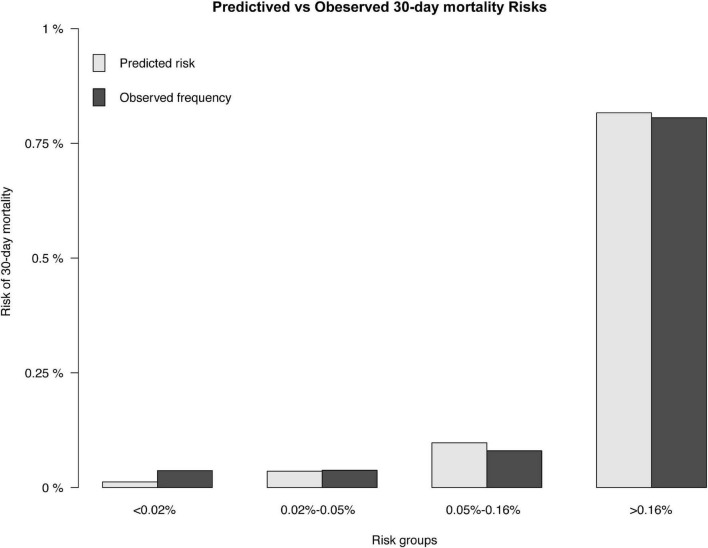
Predicted and observed 30-day mortality risk. The predicted (gray) and observed (black) risk of 30-day mortality for patients following PCI.

**FIGURE 4:**
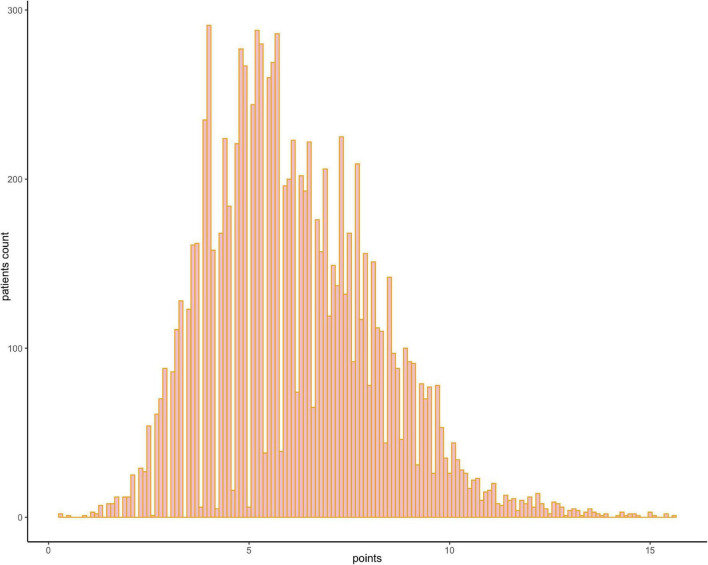
Frequency distribution bar graph of the cohort. The distribution of the risk scores of the population.

The results of the univariable logistic analysis are shown in Table 2. Variables with a *p*-value < 0.1 in the univariable logistic regression analysis, including age, male sex, prior MI, STEMI, smoking, TIMI flow 0–1 before PCI, TIMI flow 0–2 after PCI, second generation drug-eluting stent (DES), renal insufficiency, and cardiac dysfunction were selected for subsequent stepwise backward multivariable logistic analysis; variables with *p* < 0.05 were retained in the multivariable logistic regression (Table 2). Therefore, age, male sex, STEMI, TIMI flow 0–2 after PCI, and cardiac dysfunction were selected to formulate a nomogram (Figure 1 and Table 3). The average AUC generated by bootstrap resampling was 0.884. There was a good agreement between the predicted and the observed 30-day mortality risk (Figures 2, 3). The Hosmer–Lemeshow test also demonstrated that the model was a good fit (X^2^ = 10.605, *p* = 0.225). The distribution of the risk scores of the population were shown in Figure 4.

**TABLE 2:** Logistic regression model for 30-day mortality.

	Univariable		Multivariable		
		
	OR (95% CI)	*P*-value	OR (95% CI)	*P*-value	Coefficient
Age	1.12 (1.08–1.18)	<0.001	1.1 (1.05–1.15)	<0.001	0.0924
Men	0.17 (0.07–0.38)	<0.001	0.22 (0.09–0.52)	0.001	−1.5214
BMI	0.97 (0.85–1.09)	0.58			
Diabetes mellitus	0.44 (0.15–1.27)	0.13			
Hypertension	0.99 (0.44–2.23)	0.97			
Hyperlipidemia	0.52 (0.24–1.14)	0.1			
Prior PCI	1.19 (0.5–2.85)	0.7			
Prior CABG	2.1 (0.79–5.6)	0.14			
Prior Cerebrovascular disease	0.97 (0.13–7.21)	0.98			
Prior MI	3.76 (1.62–8.71)	<0.001			
STEMI	6.28 (2.86–13.79)	<0.001	5.72 (2.44–13.38)	<0.001	1.7431
NSTEMI	1.84 (0.43–7.85)	0.41			
ACS	1.28 (0.55–2.97)	0.56			
Smoking	0.35 (0.15–0.82)	0.02			
LM-LAD	0.68 (0.3–1.53)	0.35			
Multivessel disease	1.32 (0.49–3.51)	0.58			
TIMI flow 0–1 before PCI	2.43 (1.09–5.43)	0.03			
TIMI flow 0–2 after PCI	8.67 (3.44–21.85)	<0.001	8.45 (3.22–22.17)	<0.001	2.1344
First-generation DES	0.82 (0.19–3.5)	0.79			
Second-generation DES	0.35 (0.15–0.81)	0.01			
Renal insufficiency	4.87 (1.45–16.35)	0.01			
Lesion Length	1.01 (0.99–1.02)	0.49			
Cardiac dysfunction	6.6 (2.84–15.36)	<0.001	3.02 (1.2–7.62)	0.019	1.1066

Variables are shown as mean (standard deviation, SD) or n (%). PCI, percutaneous coronary intervention; CABG, coronary artery bypass grafting; ACS, acute coronary syndrome; NSTEMI, non-ST-segment elevation myocardial infarction; STEMI, ST-segment elevation myocardial infarction; LM-LAD, left main artery disease and left anterior descending artery disease; DES, drug-eluting stent; TIMI, thrombolysis in myocardial infarction. Cardiac dysfunction was defined as LVEF <50%. Renal insufficiency was defined as estimated Glomerular filtration rate (eGFR) <30 ml/min/1.73 m^2^.

**TABLE 3:** Risk-prediction nomogram.

Age (years)	Score	Sex	Score
20	0	Male	0
25	1	Female	2
			
30	1	**Cardiac dysfunction**	**Score**
			
35	2	No	0
40	3	Yes	2
			
45	3	**STEMI**	**Score**
			
50	4	No	0
55	5	Yes	3
			
60	5	**TIMI 0–2 after PCI**	**Score**
			
65	6	No	0
70	7	Yes	3
75	7		
80	8		
85	9		
90	9		
95	10		

STEMI, ST-segment elevation myocardial infarction; TIMI, thrombolysis in myocardial infarction; PCI, percutaneous coronary intervention. Cardiac dysfunction was defined as LVEF <50%.

**FIGURE 5:**
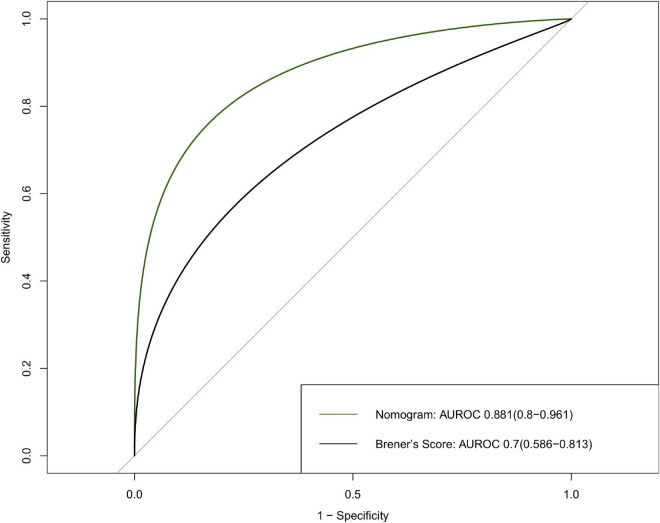
Discrimination of the 30-day mortality of derivation cohort. ROC curves of the nomogram and Brener’s risk model predicting 30-day mortality with corresponding AUC values. ROC, receiver operating characteristic; AUC, area under curve.

The nomogram had a good performance in predicting 30-day mortality after PCI, with an AUC of 0.881 (95% CI: 0.8–0.961), and the nomogram showed a better predictive performance than Brener’s model with an AUC of 0.7 (95% CI: 0.586–0.813) (Figure 5).

**FIGURE 6:**
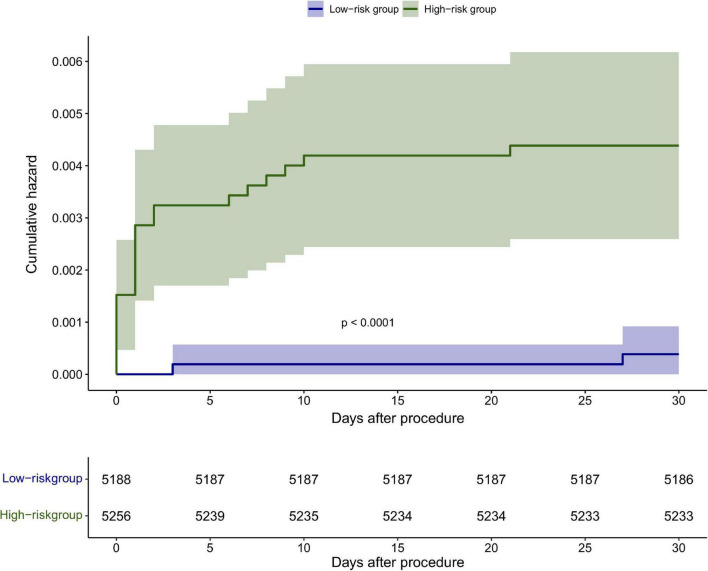
Kaplan–Meier analysis of the 30-day mortality based on the median score <4 and ≥4 in the cohort.

**FIGURE 7:**
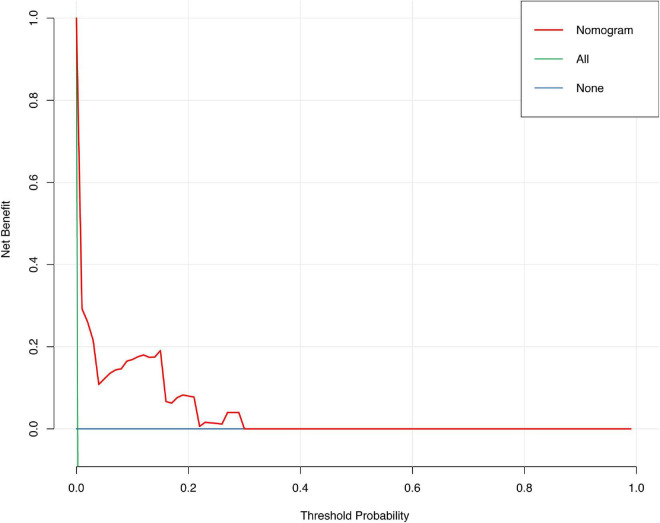
Decision carve analysis of the nomogram predicting 30-day mortality after PCI.

The discrimination of the nomogram was also assessed by Kaplan–Meier analysis (Figure 6). The patients were stratified by the median of the risk score (score = 4) of our cohort. A total of 5,188 (49.67%) patients were stratified into the low-risk group and 5,256 (50.33%) patients were stratified into the high-risk group. The cumulative probability of the occurrence of 30-day mortality was significantly higher in patients with scores higher than 4 (*p* < 0.0001). The decision curve analysis (DCA) demonstrated that the nomogram predicting 30-day mortality confers a net benefit over the “full treatment” and “no treatment” strategies in our cohort (Figure 7).

## Discussion

In the present study, we established a nomogram for predicting 30-day mortality after PCI in a large-scale, real-world PCI cohort. The nomogram shows excellent discrimination and calibration, which may help clinicians in risk prediction.

To our knowledge, this study firstly used a nomogram to predict the 30-day mortality of patients undergoing PCI. Nomogram is a graphic and easy-to-use calculation instrument which could make an individualized prediction of 30-day mortality after PCI. This nomogram incorporates clinical variables and angiographic variables: age, sex, cardiac dysfunction, STEMI, and TIMI flow after PCI. Previous scoring systems rarely incorporated angiographic variables (6, 15, 16).

In Brener’s risk model, cardiac function and renal function were not included due to the lack of data (9). However, in previous studies, cardiac function and renal insufficiency are independent risk factors of 30-day mortality after PCI (3, 17–19), and both are included in several risk scores, such as the CADILLAC score (17), the New York State Risk Score (20), and Mayo Clinic Risk Score (5). To improve the predictive value of the risk score, left ventricular ejection fraction and renal function are added to our analysis. Finally, cardiac dysfunction was selected to incorporate the nomogram. Compared to Brener’s score, this nomogram exhibited an enhanced 30-day mortality predictive value in Asian patients (0.7 vs. 0.881). Among the total patients undergoing PCI with a score of <4, 0.03% died within 30 days; while for those with scores of ≥4, 0.36% died within 30 days after PCI. The results showed that the risk of mortality increased as the sum of scores increased.

Previous studies established several risk models for predicting 30-day mortality after PCI, including the New York State risk score (20), the British Columbia PCI risk score (6), the ALPHA score (21), and the Mayo Clinic risk score (5); all of these scores focused on the European or American population. Our study is the first risk model aimed to predict the 30-day mortality of Asian patients undergoing PCI.

In conclusion, we established a graphic nomogram to predict 30-day mortality in patients who underwent PCI based on a large-scale real-world cohort study, and our nomogram exhibited better performance than Brener’s score. This nomogram would be a good tool to help doctors identify patients with high risk and make better clinical decisions.

## Data availability statement

The original contributions presented in this study are included in the article, further inquiries can be directed to the corresponding author.

## Ethics statement

This study was reviewed and approved by the Institutional Review Board of the Fuwai Hospital, Beijing, China. Written informed consent was obtained from all participants for their participation in this study.

## Author contributions

JS, YL, and WW participated in the research design. JS, YL, JC, JY, JW, and JG performed the data analysis and interpretation. JS and YL drafted the manuscript. Y-DT edited the manuscript and supervised the studies. All authors read and approved the final manuscript.

## Footnotes

1
https://www.r-project.org/

## Abbreviations

PCI: percutaneous coronary intervention
DES: drug-eluting stents
LAD: left anterior descending coronary artery
LM: left main coronary artery
TIMI: Thrombolysis in Myocardial Infarction
STEMI: ST-segment elevation myocardial infarction.
